# Assessment of Cardiovascular Risk in Women with Periodontal Diseases According to C-reactive Protein Levels

**DOI:** 10.3390/biom11081238

**Published:** 2021-08-19

**Authors:** Claudia Da Venezia, Nayib Hussein, Marcela Hernández, Johanna Contreras, Alicia Morales, Macarena Valdés, Francisca Rojas, Loreto Matamala, Patricia Hernández-Ríos

**Affiliations:** 1 Faculty of Dentistry, University of Chile, Santiago 8380544, Chile; claudia.davenezia@ug.uchile.cl (C.D.V.); nayib.hussein@ug.uchile.cl (N.H.); frojas@odontologia.uchile.cl (F.R.); 2 Laboratory of Periodontal Biology, Faculty of Dentistry, University of Chile, Santiago 8380544, Chile; mhernandezrios@gmail.com; 3 Department of Pathology and Oral Medicine, Faculty of Dentistry, University of Chile, Santiago 8380544, Chile; 4 Department of Conservative Dentistry, Faculty of Dentistry, University of Chile, Santiago 8380544, Chile; jcontreras@odontologia.uchile.cl (J.C.); amorales@odontologia.uchile.cl (A.M.); lmatamalalopez@odontologia.uchile.cl (L.M.); 5 Center for Epidemiology and Surveillance of Oral Diseases (CESOD), Faculty of Dentistry, University of Chile, Santiago 8420000, Chile; 6 Faculty of Medicine, School of Public Health, University of Chile, Santiago 8380453, Chile; macavaldes@uchile.cl; 7 Center for Climate and Resilience Research CR2, University of Chile, Santiago 8370449, Chile

**Keywords:** cardiovascular disease, periodontal diseases, non-communicable diseases, C-reactive protein, gingival crevicular fluid, periodontitis, gingivitis, women, cardiovascular risk, biomediators

## Abstract

Cardiovascular diseases (CVD) are highly prevalent non-communicable diseases worldwide. Periodontitis may act as a non-traditional cardiovascular risk (CVR) factor, linked by a low-grade systemic inflammation mediated by C-reactive protein (CRP). Patients with periodontitis reported higher serum CRP levels; however, a CRP systemic and periodontal correlation in gingival crevicular fluid (GCF) and its CVR impact have been barely studied. We aimed to assess the association between periodontal diseases and CVR in a group of adult women, based on serum high-sensitivity CRP (hs-CRP) levels; and secondly, to determine the association between serum and GCF CRP levels. Gingival crevicular fluid and blood samples were obtained from women with periodontitis, gingivitis, and healthy controls. Serum and GCF CRP were determined by turbidimetric method and Luminex technology, respectively. Data were analyzed and adjusted by CVR factors. All women presented moderate CVR, without an evident association between serum hs-CRP levels and periodontal diseases. While serum hs-CRP concentrations did not significantly differ between groups, patients with gingivitis and periodontitis showed higher CRP levels in GCF, which positively correlated to CRP detection in serum.

## 1. Introduction

Cardiovascular diseases (CVD) are a group of diseases that affect the heart and blood vessels. Their high prevalence positions them as the leading cause of death globally, especially those associated with atherosclerotic processes [1]. Traditional risk factors include age, high total cholesterol or high low-density lipoprotein (LDL), low level of high-density lipoprotein (HDL), high blood pressure, diabetes mellitus, obesity, smoking, family history of CVD, low socioeconomic status, and sex [2,3]. Even when men are the most common sufferers, CVD also have high relative relevance in women’s death. It is projected that around 50% of the female population will die from CVD, which sets up these diseases as one of the main causes of death, especially for women of younger ages. They are particularly affected, presenting higher in-hospital mortality and hospital readmission than men of the same age [4,5].

C-reactive protein (CRP) is an acute-phase reactant produced primarily in the liver that represents the prototype marker of systemic inflammation, which is elevated in many chronic non-communicable diseases [1,6]. Besides, it takes part in the pathogenesis of atherosclerosis, participating in the formation and rupture of atheroma plaques [5]. CRP is currently a validated predictor of future cardiovascular events [7], with three categories of relative CVD risk, according to high-sensitivity (hs) CRP serum concentrations: low risk (<1.0 mg/L), moderate risk (1.0 to 3.0 mg/L), and high risk (>3.0 mg/L) of CVD [8].

Periodontal diseases, such as gingivitis and periodontitis, are chronic inflammatory diseases triggered by oral biofilm. While gingivitis is mainly characterized by gingival inflammation, periodontitis results in the progressive destruction of the tooth-supporting apparatus [9,10]. Systematic reviews and meta-analyses have considered periodontitis as an independent and non-traditional CVD risk factor, particularly in adults under 45 years old and middle-aged women [11,12]. Low-grade systemic inflammation may link both diseases [13], and hs-CRP might become a marker of inflammatory burden and systemic risk impact that could be included in the periodontal classification framework in the future [14]. Different studies consistently reported higher hs-CRP serum levels in periodontitis and gingivitis patients compared to healthy controls, showing a direct correlation with periodontal inflammation degree [15,16].

Since the 1990s, CRP has also been detected locally in gingival crevicular fluid (GCF), a serum exudate that leaks through the gingival sulcus and contains blood molecular and cellular components [16]. Some studies found higher CRP levels in GCF of periodontitis patients compared to healthy individuals, but the evidence is scarce and limited, especially regarding gingivitis patients [17,18]. Thereby, if CRP levels in GCF were correlated with its serum concentration, a new, simple, and non-invasive periodontal link to determine systemic inflammation, and even cardiovascular assessment, could be eventually considered and developed [19].

This study aimed to assess the association between periodontal diseases and cardiovascular risk in a group of adult women, based on serum hs-CRP levels; and secondly, to determine the association between serum and gingival crevicular fluid CRP levels.

## 2. Materials and Methods

### 2.1. Design and Sample Calculation

A quantitative, analytical, and cross-sectional study. The primary outcome was defined by hs-CRP serum concentrations ≥3 mg/L. According to previous data [20], considering a confidence interval of 95%, a statistical power of 80%, and a two-tailed test, 111 individuals were required for the study (37 participants per group).

### 2.2. Study Population

Chilean women between 18 and 44 years of age and at least 12 teeth in the mouth were recruited at the Dental Clinic of the Faculty of Dentistry, University of Chile, between 2017 and 2019. A convenience sampling technique was used. 

Exclusion criteria comprehended individual history of CVD; atherosclerosis; high blood pressure (hypertension); diabetes mellitus; acute inflammatory diseases; previous periodontal treatment; the presence of concomitant apical periodontitis; pregnant women; and anti-inflammatory, antibiotic, or immunomodulatory therapy within the previous six months. 

The study protocol was approved by the Institutional Biosafety Board and Ethics Committee of the Faculty of Dentistry of the University of Chile(inform code 2017/02; date of approval: 2 May 2017). Written informed consent was obtained from all volunteers, according to the guidelines of the Declaration of Helsinki.

### 2.3. Physical Examination and Clinical Laboratory Measurements

A thorough medical history was obtained for all study participants, including self-reported cardiovascular risk factors (age, educational level, smoking, hypertension, and diabetes). Weight, height, body mass index (BMI), and pressure measurements were registered by a calibrated operator.

Fasting blood samples were obtained through a puncture in the antecubital vein, and the samples were sent to the clinical laboratory of the University Hospital to determine serum values of glycated hemoglobin (HbA1c), hs-CRP, and lipids (total cholesterol, HDL, LDL, and triglycerides).

### 2.4. Clinical Periodontal Examination

All participants underwent a full set of periapical radiographs and a periodontal clinical examination was performed by two calibrated examiners (kappa ≥ 80%). Using a North Carolina periodontal probe UNC-15 (Hu-Friedy, Chicago, IL, USA), the gingival index (GI) was recorded at 4 sites per tooth (mesiobuccal, buccal, distobuccal, and lingual or palatine), while bleeding on probing (BOP), probing depth (PD), and clinical attachment loss (AL) were measured at 6 sites per tooth (mesiobuccal, buccal, distobuccal; mesiolingual, lingual or palatine, and distolingual). BOP was recorded as present or absent, after 15 s. PD was recorded as the distance from the gingival margin to the base of the gingivo-dental sulcus, and AL was defined as the distance from the amelo-cemental junction to the base of the gingivo-dental sulcus.

Women were classified according to their periodontal status in: healthy (PD < 4 mm, GI < 1); gingivitis (PD < 4 mm, GI ≥ 1); and periodontitis (≥ 5 teeth with PD ≥ 4 mm, AL ≥ 1 mm, BOP > 20%) [18,21].

### 2.5. Gingival Crevicular Fluid Collection

Gingival crevicular fluid samples were obtained by two calibrated operators, from 4 sites per participant: the site with the highest PD in each quadrant, in women with periodontitis; the site with the most severe GI per quadrant in the gingivitis group; and from one site with PD < 4 mm and no bleeding on probing, in each quadrant of healthy participants.

The GCF collection method was performed according to the study of Hernández Rios et al. in 2009 [22]. Briefly, after isolating the tooth with cotton rolls, the supragingival plaque was manually removed, without touching the marginal gingiva. Sites were gently air-dried with a triple syringe, and GCF was collected with paper strips (Periopaper, ProFlow, Amityville, NY, USA), placed into the gingival crevice until a slight resistance was felt, for 30 s. Contaminated strips with blood or saliva were excluded.

The fluid was eluted and stored at −80 °C until analysis. CRP levels were determined with a Multiplex immunoassay (Magnetic Luminex Assay, R&D Systems, Minneapolis, MN, USA) on a Luminex platform (Magpix, Millipore, St Charles, MO, USA), according to the manufacturer’s recommendations. The results were expressed in pg of PCR/mL of elution.

### 2.6. Definition of Cardiovascular Risk Factors and Outcome Variables

The primary outcome variable was defined as the presence of hs-CRP > 3 mg/L (high cardiovascular risk). Secondarily, the presence of hs-CRP ≥ 1 mg/L (moderate-high cardiovascular risk) and CRP levels in GCF (pg/mL) were analyzed.

Dyslipidemia was reported as total cholesterol ≥ 200 mg/dL, HDL < 40 mg/dL, LDL ≥ 130 mg/dL, or triglycerides ≥ 150 mg/dL [23]. Overweight was considered when BMI was ≥25 and <30 kg/m^2^, and obesity with BMI ≥ 30 kg/m^2^ [24]. High blood pressure was recorded when the mean systolic blood pressure was ≥140 mmHg and/or the mean diastolic blood pressure was ≥90 mmHg. Smoking was dichotomously categorized in smokers or non-smokers, and HbA1c values, age, and educational level were also considered.

### 2.7. Statistical Analysis

The variables were described using absolute or relative frequencies (percentages), or medians and interquartile ranges. The Shapiro–Wilk test was used to test normality in continuous variables and the association between categorical variables was analyzed using the Chi-square test. Serum CRP and GCF levels between the three study groups were analyzed using the K-Wallis test and Spearman’s correlation.

The association between periodontal disease (gingivitis and periodontitis) and cardiovascular risk was determined using serum CRP ≥ 1 mg/L (moderate to high cardiovascular risk) or serum CRP > 3 mg/L (high cardiovascular risk) as outcome variables according to the ACCF/AHA guidelines 2010. Bivariate and multivariate logistic regression analyses were performed to adjust for potential confounders. Models were constructed by entering covariates adjustment progressively, starting with demographic variables and both oral and classical cardiovascular risk factors, and stepwise adjustment was applied. Finally, the model was adjusted by BMI as it was identified as a confounding variable. The information was analyzed with a statistical package (STATA 12, StataCorp, College Station, TX, USA).

## 3. Results

While 130 women under 45 years attended the evaluation, 114 of them met the eligibility criteria. However, 2 women did not attend the blood and GCF sampling session, so 112 volunteers were finally included.

Demographic, clinical, and hematological parameters of the groups with gingivitis (*n* = 39), periodontitis (*n* = 36), and healthy controls (*n* = 37) are shown in Table 1. Age median was significantly higher in individuals with periodontitis than in gingivitis (29.1 [5.9] and 26.2 [6.3] years, respectively) (*p* < 0.008); while BMI medians progressively increased from health to gingivitis and periodontitis (21.2 [2.9], 23.8 [4.3], 24.4 [6.3] kg/m^2^, respectively), with significant differences between the group of gingivitis and the controls (*p* < 0.008). The glycated hemoglobin median was slightly higher in volunteers affected by periodontitis (5.3% [0.3]), showing statistical differences with the group of gingivitis (5.1% [0.4]) (*p* < 0.008) Meanwhile, educational level, smoking habit, high blood pressure, and dyslipidemia were homogeneously distributed between groups (*p* > 0.05). 

In relation to periodontal clinical parameters, gingival index (GI) and bleeding on probing (BOP) were significantly higher in gingivitis (GI: 1.1 [0.3]; BOP: 33.9% [11.3]) and periodontitis (GI: 1.0 [0.3]; BOP: 40.4% [24.5]), in relation to controls (GI: 0.6 [0.5]; BOP: 17.9% [15.6]) (*p* < 0.008). Probing depth (PD) and clinical attachment loss (AL) medians were significantly elevated in individuals affected by periodontitis (PD: 2.47 [0.29] mm; AL: 0.99 [0.65] mm), in comparison to those with gingivitis (PD: 2.06 [0.23] mm; AL: 0.39 [0.39] mm) (*p* < 0.008) or the controls (PD: 2.07 [0.25] mm; AL: 0.56 [0.5] mm) (*p* < 0.008) (Table 1).

Serum and gingival crevicular fluid CRP levels of study participants are shown in Table 2. Even though the medians of serum hs-CRP levels were higher in periodontitis (median: 1.68 [2.9] mg/L) than in gingivitis (median: 1.30 [2.2] mg/L) or healthy women (median: 1.25 [3.2] mg/L), the differences did not reach statistical significance (*p* = 0.82). Median CRP levels in gingival crevicular fluid, on the other hand, were significantly raised in gingivitis (1178.2 [2882.5] pg/mL) and periodontitis participants (1131.2 [3667.6] pg/mL) in comparison to healthy women (157 [398.0] pg/mL) (*p* < 0.001).

A positive and statistically significant association between CRP in serum and GCF was observed in all groups (*p* = 0.00), with a Spearman’s rank correlation coefficient (rho) of 0.87, 0.63, 0.88, and 0.70 in healthy individuals, gingivitis, periodontitis, and the total sample of volunteers, respectively (Table 3). However, an association between periodontal disease (gingivitis and periodontitis) and moderate to high (serum hs-CRP ≥ 1 mg/L) or high (serum hs-CRP > 3 mg/L) cardiovascular risk was not evident in adjusted bivariate (Table 4, models 1.a and 1.b; *p* > 0.05) nor multivariate logistic models (Table 4, models 2.a and 2.b; *p* > 0.05). As it is seen in model 2c, body mass index was the single covariate that presented a significant association with moderate to high (*p* = 0.01, CI: 1.09–1.46) and high (*p* = 0.04, CI: 1.0–1.21) cardiovascular risk. The age and glycated hemoglobin covariates did not present a significant association with any of the variables studied (*p* > 0.05) (data not shown).

## 4. Discussion

Cardiovascular diseases, especially those derived from atherosclerotic processes, have become an increasingly relevant public health problem. Serum hs-CRP, a validated inflammatory marker for cardiovascular risk stratification, has been raised in some periodontal patients, representing a possible link between both chronic non-communicable diseases [1,6].

In the current study, we were not able to confirm an association between hs-CRP-based risk stratification and periodontal diseases in adult Chilean women; however, we found a strong association between CRP levels in serum and GCF, especially in periodontitis, revealing its potential usefulness as a surrogate non-invasive screening method for systemic inflammation. Although medians of CRP serum concentrations were slightly higher in periodontitis than in gingivitis or healthy participants, no statistically significant differences were found between the study groups. These results are in disagreement with previous studies that reported lower serum CRP values in healthy individuals compared with periodontitis and/or gingivitis groups of individuals [15,25].

The discrepancies between this study and those reported in the literature may be attributable, at least in part, to differences in the clinical expression of periodontal disease. The presence of generalized or severer forms of periodontitis, with higher levels of destruction and chronicity, may trigger a greater systemic inflammation and higher levels of CRP in the blood [18]. However, the participants of this study presented a lower severity and extent of periodontitis when analyzing clinical parameters and comparing them with other investigations [15,16,25]. This might be probably explained by the inclusion criteria, in concordance with the milder forms of periodontal diseases that are epidemically associated with the young age, as well as a fairly high educational level [9].

The present study did not show a significant association between gingivitis or periodontitis and CVD risk, in the bivariate or multivariate regression analyses. Just a few studies associate periodontal diseases with cardiovascular risk, and they do not usually include a gingivitis group or adjust by confounding factors. Recent research found a significant association between severe periodontitis and moderate CV risk (CRP 1–3 mg/L) [26]. Another publication also reported a relationship between moderate to severe periodontitis and high cardiovascular risk (CRP > 3 mg/L), but it was lost after adjustment for CVD risk factors (sex, age, educational level, smoking, BMI, diabetes, and HDL cholesterol) [27]. Indeed, a systematic review reported that a low number of studies controlled for potential confounders, suggesting that the combination of these variables with periodontitis could result in moderately elevated levels of CRP, contributing to an increased risk of CVD [28]. 

Between the measured and adjusted parameters of the present investigation, educational level, smoking habit, high blood pressure, and dyslipidemia did not show significant differences between groups, nor interfered in the results of our report. BMI, on the other hand, became especially relevant.

High BMI and obesity are increasingly prevalent global health problems [29] that reach more than one-third of the Chilean population [30]. They might be able to accelerate the atherosclerosis progression through the induction of a prothrombotic and proinflammatory state [31]. The secretion of inflammatory mediators (such as tumor necrosis factor-alpha and interleukin-6) by adipocytes stimulates CRP releasing and, in parallel, contributes to periodontal tissue destruction [32,33]. While many studies associating periodontal diseases with higher serum CRP levels do not measure or control BMI [15,16,18,34,35], obesity and overweight were adjusted and not excluded from our study because of their relevance and weight on external validity. Even when BMI presented significant differences between gingivitis subjects and healthy individuals, it was the only variable that influenced the serum hs-CRP increase and was significantly associated with moderate and high CVD risk in both periodontal disease groups. Based on the results of this study, the burden of milder forms of periodontal disease over serum CRP levels from high BMI individuals is probably irrelevant, which could be a interesting finding to consider, and opens a field for future studies.

Diabetes mellitus is a classic risk factor that also affects both periodontal and cardiovascular diseases. It can trigger a systemic inflammatory process with chronic elevation of inflammatory mediators and CRP [36]. Diabetic patients were excluded from this research, and although glycosylated hemoglobin levels were slightly different between groups, they remained within normal values in all participants (less than 5.7%).

Age was another heterogeneous factor between the groups, which was higher in periodontitis participants. With increasing age, the prevalence of cumulative periodontal damage and the prevalence of high CVD risk increases [9,37]. To shelter participants’ safety and include a lower prevalence of comorbidities and confounders, this study only considered adult women under 45 years old, a limited range that did not associate with cardiovascular risk in our results (*p* > 0.05). 

Ethnicity and gender are other determinants that may affect CRP levels and may help to explain the differences with other investigations, carried out in mixed populations [16]. In a longitudinal study, periodontitis was associated with increased serum CRP levels and CVD mortality in men, but not in women [38]. A gender influence on serum CRP measurements in periodontal disease was evidenced, with an association between CRP and probing depths mainly in men, and between overweight and obesity mainly in women. Similar to our findings, female adiposity was able to overcome the impact of periodontal status in serum PCR levels [39], while CRP GCF levels did not show significant differences between both sexes [17]. 

The representation of a population restricted to young adult women is especially relevant in young adults because of the lack of accumulation of other risk factors that normally occur in older individuals [10,11,21]. Furthermore, considering the particularities of women in the interrelation of cardiovascular and periodontal diseases, this topic is underrepresented in scientific reports, and it would be interesting to explore it further. The female population of this study was selected given the particular impact of CVD over young women, setting aside the influence of male gender as a confounding factor in cardiovascular risk. It constitutes, namely, the first report of its kind in Latin American women, contributing to the scant worldwide evidence about the serum and local CRP levels associated with cardiovascular risk in periodontal diseases.

In general, the literature barely describes GCF CRP variations in gingivitis and periodontitis patients. Gingivitis and periodontitis are different stages of the periodontal inflammatory and destructive process, which involve the synthesis of different immune mediators. C-reactive protein raised in serum upon interleukin-1 exposure (a cytokine involved in bone resorption), while CRP levels might be locally affected by differences in periodontal disease progression and activity, due to variations in the polymorphonuclear neutrophil number and the gingival fluid volume measured in the crevice [18]. Some authors reported significantly higher CRP levels in the gingival crevicular fluid of individuals with periodontitis, as compared to healthy controls, but not in the gingivitis group [18,25]. In the current study, we found statistically higher CRP levels in GCF from individuals affected by both periodontitis and gingivitis, compared to healthy ones. Considering the lack of an evident association between periodontal disease and serum CRP levels found in our study, the aforementioned fact might be explained, to some extent, by a local production of CRP within periodontal tissues, which has been previously reported and demonstrated [19,40]. However, moderate to strong positive correlations between the concentrations of this protein in GCF and serum were also evidenced in all study groups, showing that CRP measurements in GCF may reflect its serum levels, as demonstrated in past works [18,25]. This fact suggests that CRP in GCF might be indicative of systemic inflammation, either generated at distant organs and/or induced by periodontal disease [19].

The finding of a local–systemic correlation is interesting. The gingival crevicular fluid collection might eventually become a simple and non-invasive tool to link periodontal and general status, aid in the detection of systemic inflammation [19], and help in the assessment and monitoring of cardiovascular and other chronic non-communicable diseases. However, further research is needed. Deepening the links between periodontal diseases and C-reactive protein could contribute to the subsequent prevention and control of prevalent inflammatory diseases, placing special emphasis on timely periodontal care that may positively influence public health. 

Future studies should consider larger and more heterogeneous samples of multicentric populations with different severities of periodontal disease, a greater number of assorted risk and confounding factors, and increased control over them.

## 5. Conclusions

All women had moderate cardiovascular risk, without an evident association between serum hs-CRP levels and periodontal diseases. Patients with gingivitis and periodontitis showed higher levels of CRP in gingival crevicular fluid, which positively correlated to its detection in serum.

## Figures and Tables

**Table 1:** Demographic, clinical, and hematological parameters of study participants.

Variable	Healthy (*n* = 37)	Gingivitis (*n* = 39)	Periodontitis (*n* = 36)	*p*-Value
Age [years, median (IQR)]	26.3 (5)	26.2 (6.3) *	29.1 (5.9)	0.02
Educational Level (median)	Tertiary Education	Tertiary Education	Tertiary Education	0.08
Smoking habit [*n* (%)]	9 (24.3%)	9 (23%)	11 (30.5%)	0.73
BMI [kg/m^2^, median (IQR)]	21.2 (2.9)	23.8 (4.3) **	24.4 (6.3)	0.00
HBP [*n* (%)]	0	0	2 (5.6%)	0.11
Dyslipidemia [*n* (%)]	10 (27%)	8 (20.5%)	14 (38.9%)	0.20
HbA1c [%, median (IQR)]	5.2 (0.1)	5.1 (0.4) *	5.3 (0.3)	0.01
GI [median (IQR)]	0.6 (0.5)	1.1 (0.3) **	1.0 (0.3) **	0.00
BOP [%, median (IQR)]	17.9 (15.6)	33.9 (11.3) **	40.4 (24.5) **	0.00
PD [mm, median (IQR)]	2.07 (0.25)	2.06 (0.23) *	2.47 (0.29) **	0.00
AL [mm, median (IQR)]	0.56 (0.5)	0.39 (0.39) *	0.99 (0.65) **	0.00

Values were expressed in median (interquartile range) and absolute frequencies (%). IQR: interquartile range, BMI: body mass index, HBP: high blood pressure, HbA1c: glycated hemoglobin. GI: gingival index, BOP: bleeding on probing, PD: probing depth, AL: clinical attachment loss. * *p* < 0.008 (Kruskal–Wallis post hoc test) compared with periodontitis group. ** *p* < 0.008 (Kruskal–Wallis post hoc test) compared with the healthy group.

**Table 2:** C-reactive protein levels in serum and gingival crevicular fluid of study participants.

Group	Serum hs-CRP mg/L	GCF CRP pg/mL
	Median (IQR)	Median (IQR)
Healthy	1.25 (3.2)	157 (398.0)
Gingivitis	1.30 (2.2)	1178.2 (2882.5) *
Periodontitis	1.68 (2.9)	1131.2 (3667.6) *
*p*-value	0.82	<0.001

CRP: C-Reactive protein, GCF: gingival crevicular fluid, IQR: interquartile range. * *p*-value < 0.008 (Kruskal–Wallis post hoc test) compared with the healthy group.

**Table 3:** C-reactive protein correlations between serum and gingival crevicular fluid.

Group	*n*	Spearman’s Correlation (rho)	*p*-Value
Healthy	37	0.87	0.00
Gingivitis	39	0.63	0.00
Periodontitis	36	0.88	0.00
Total	112	0.70	0.00

**Table 4:** Association between periodontal disease and cardiovascular risk, determined by serum CRP ≥ 1 mg/L (moderate to high cardiovascular risk) or serum CRP > 3 mg/L (high cardiovascular risk).

Model	CRP ≥ 1 mg/L			CRP > 3 mg/L		
	OR	*p*-Value	CI	OR	*p*-Value	CI
Model 1.a	1.36	0.5	0.54–3.38	0.53	0.24	0.19–1.51
Model 1.b	1.5	0.39	0.58–3.84	0.91	0.86	0.34–2.46
Model 2.a	0.85	0.75	0.31–2.29	0.4	0.1	0.13–1.21
Model 2.b	0.99	0.98	0.35–2.75	0.71	0.52	0.25–2.01
Model 2.c	1.26	0.01	1.09–1.46	1.1	0.04	1.0–1.21

CRP: C- reactive protein, OR: odds ratio, CI: confidence interval. a: gingivitis group; b: periodontitis group; c: covariate body mass index. Model 1: bivariate regression analysis. Model 2: multivariate regression analysis adjusted by body mass index.

## Data Availability

Not applicable.
